# Single-Molecule Counting of Point Mutations by Transient DNA Binding

**DOI:** 10.1038/srep43824

**Published:** 2017-03-06

**Authors:** Xin Su, Lidan Li, Shanshan Wang, Dandan Hao, Lei Wang, Changyuan Yu

**Affiliations:** 1 College of Life Science and Technology, Beijing University of Chemical Technology, Beijing, 100029, China; 2 Institute of Quality Standard and Testing Technology for Agro-Products, Chinese Academy of Agricultural Sciences, Beijing, 100081, China

## Abstract

High-confidence detection of point mutations is important for disease diagnosis and clinical practice. Hybridization probes are extensively used, but are hindered by their poor single-nucleotide selectivity. Shortening the length of DNA hybridization probes weakens the stability of the probe-target duplex, leading to transient binding between complementary sequences. The kinetics of probe-target binding events are highly dependent on the number of complementary base pairs. Here, we present a single-molecule assay for point mutation detection based on transient DNA binding and use of total internal reflection fluorescence microscopy. Statistical analysis of single-molecule kinetics enabled us to effectively discriminate between wild type DNA sequences and single-nucleotide variants at the single-molecule level. A higher single-nucleotide discrimination is achieved than in our previous work by optimizing the assay conditions, which is guided by statistical modeling of kinetics with a gamma distribution. The KRAS c.34 A mutation can be clearly differentiated from the wild type sequence (KRAS c.34 G) at a relative abundance as low as 0.01% mutant to WT. To demonstrate the feasibility of this method for analysis of clinically relevant biological samples, we used this technology to detect mutations in single-stranded DNA generated from asymmetric RT-PCR of mRNA from two cancer cell lines.

Due to the diagnostic significance of single-point mutations and single-nucleotide variants (SNV) in the human genome, there is an urgent need to develop high-confidence SNV identification methods^1^,^2^. Current efforts have focused on enhancing single-nucleotide selectivity, including the development of digital PCR^3^, barcode-based assays^4^, nanopore approaches^5^ and next-generation sequencing^6^. Hybridization probes^7^ (such as molecular beacons, binary probes, and artificial modified probes) effectively detect mutations in DNA sequences where the corresponding wild type and mutant alleles are known. The specificity of these probes is dependent on their hybridization thermodynamics, rendering it typically poor at room temperature. Recently, a simulation-guided probe pairs strategy has greatly improved the performance of hybridization probes and achieved high single-nucleotide selectivity^8^. However, the identification and quantification of SNVs in complex samples still relies on the observation of bulk fluorescence responses, which often leads to false-positive signals and poor reproducibility.

Single-molecule fluorescence techniques have substantially advanced our understanding of molecular and cellular processes over the last two decades^9^,^10^. By taking advantage of the fluorescence excitation geometry of the evanescent field in total internal reflection fluorescence microscopy (TIRFM)^11^, researchers can capture DNA targets on slides and detect single molecules of nucleic acid *in vitro*^12^,^13^. However, these methods still discriminate poorly between SNVs and wild type (WT) sequences, due to poor hybridization specificity.

When fluorescently labeled DNA oligonucleotides (strands) transiently bind to complementary ‘docking’ DNA strands immobilized on TIRFM slides, stochastic switching between fluorescent on- and off-states occurs^14^. Diffusing strands are specifically illuminated in the bound state, whereas fluorophores of unbound probes are (1) not excited as strongly if they are far from the surface, due to the exponential decay of the evanescent field intensity, and (2) even if excited, are diffusing too rapidly to generate a localized fluorescent signal on the timescale of measurement. By employing DNA transient binding, DNA-PAINT (a variation of point accumulation for imaging in nanoscale topography^15^) has been developed for simple and easy-to-implement multiplexed super-resolution imaging^16^,^17^. Inspired by DNA-PAINT, a kinetic fingerprinting approach was developed for amplification-free detection of single, unlabeled miRNA molecules with high sensitivity and specificity^18^. This approach detects the presence of point mutations and provides quantitative evaluation of SNV/WT discrimination, however, not in mixed samples which is critical for developing a useful tool for clinical diagnosis.

Herein, we describe a single-molecule approach that can detect and quantitatively evaluate point mutations by utilizing TIRFM to visualize transient DNA binding. The differential binding kinetics of SNVs relative to WT DNA molecules enables us to identify SNVs at the single-molecule level. Compared to previous work^18^, the single-nucleotide discrimination capability is further improved by optimizing the assay conditions which is guided by statistical modeling of kinetics with a gamma distribution. This kinetic approach can detect synthetic DNA with an SNV at an allelic frequency as low as 0.01% in the presence of WT. This method was successfully used to detect mutations in single-stranded DNA that was reverse-transcribed from cellular mRNA from two cancer cell lines.

## Results

### Principles of single-molecule counting of point mutations

**Figure 1:**
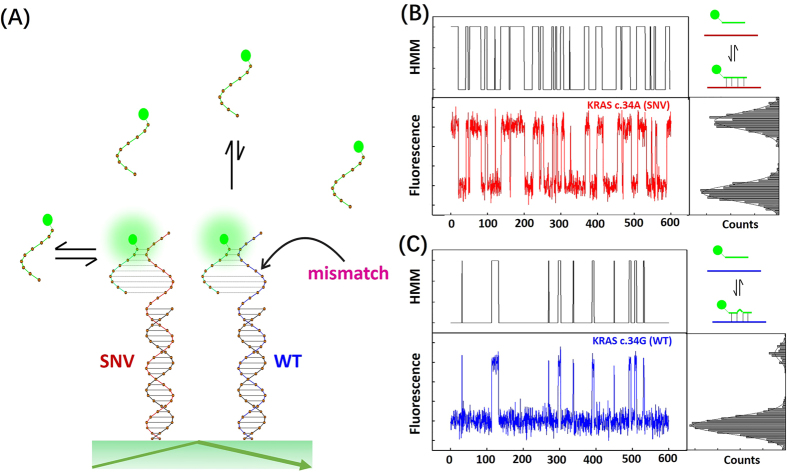
(**A**) Single-molecule counting of point mutation-containing DNA on TIRFM. WT and SNV DNA sequences can be distinguished by the distinct kinetics of transient binding with short fluorescent DNA probes. Red and blue sequences represent SNV and WT DNA molecules that only differ by one nucleotide. The fluorescent probe is complementary to the SNV target DNA, but forms a single mismatch with the WT target. (**B,C**) Single-molecule characterization of the transient binding of SNV (**B**) and WT (**C**) DNA with the short fluorescent probe, visualized using total internal reflection fluorescence microscopy, where the probe and target concentrations are 25 nM and 200 fM, respectively, and the NaCl concentration is 500 mM. Raw time trajectories (below; 10 min, 1200 frames) and idealized traces (above) predicted by hidden Markov modeling of single-molecule fluorescence reveal longer t_bound and shorter t_unbound for the SNV target relative to WT. Fluorescence intensity histograms show a higher percent (40.4%) of probes in the bound state for the mutant target than for wild type target (8.5%).

We used a gene fragment (single-stranded DNA, 39-nt) containing the KRAS c.34 G > A mutation as a model target. This G > A mutation is typically poorly detected by hybridization-based probes, because a T:G mismatch (known as a “wobble pair”) can only slightly destabilize the DNA duplex^19^,^20^. The principle of our single-molecule assay is depicted in Fig. 1A. A biotinylated capture probe (25-nt), which is complementary to the common DNA sequence shared by the SNV (KRAS c.34 A) and the WT allele (KRAS c.34 G), was immobilized on the slide surface through biotin-streptavidin interaction. Target DNA strands can be captured on the surface through stable hybridization (25-bp). A short fluorescent DNA probe that is fully complementary to the SNV, but which forms a single mismatch with WT DNA, is then added. The binding kinetics for probes of ~6–12 nt are highly sensitive to the number of complementary bases between the probe and target^21^,^22^, and are particularly sensitive to base mismatches in the middle of the probe-target duplex^21^. Upon the addition of the short probe, the SNV and WT DNA molecules yield distinct binding kinetics and fluorescence trajectories (Fig. 1B and C). The transient binding of the fluorescent probe with the SNV target creates a longer dwell time in the bound state (t_bound) and shorter dwell time of unbound state (t_unbound). The fluorescence intensity histograms shown in Fig. 1B and C are the results of statistical analysis of all molecules, suggesting that the duplex of WT DNA-probe is much less stable than that of SNV-probe.

**Figure 2:**
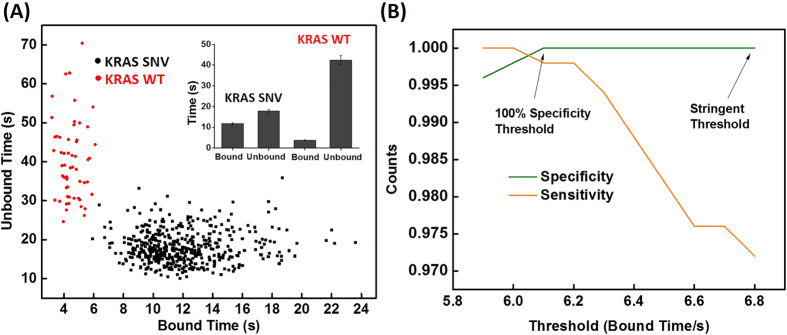
(**A**) Dwell-time mapping enables the absolute discrimination of SNV vs WT DNA. Note that the non-nucleic acid background molecules in the two groups were removed using the universal threshold (Supplementary Fig. S1). Inset: the average bound and unbound time of SNV and WT DNA. (**B**) The specificity and sensitivity of the assay for KRAS c.34 A mutation detection at varying values of the bound-state lifetime threshold. The threshold for 100% specificity is 6.1 s referring from (**A**). A more stringent threshold was set to 6.8 s by considering the derivation of the maximal t_bound of WT, threshold ≥ t_bound_max_ + 3 × std (t_bound_max_).

The dwell-time distributions of probe binding to SNV and WT DNA are shown in Fig. 2A. All of the molecules reported here exceed the universal background threshold (a number of binding + dissociation events per molecule, Supplementary Fig. S1). There are two distinctly separate clusters that represent SNV and WT DNA binding kinetics, respectively. We evaluated our assay’s performance in terms of specificity and sensitivity to determine a dwell-time threshold which would produce very accurate SNV detection. As shown in Fig. 2B, if the dwell-time threshold is set to 6.1 s, the accuracy remains at 100%, while sensitivity only decreases by 0.2%. Considering the derivation of the maximal t_bound of WT, we chose to apply a more stringent threshold (6.8 s) to achieve higher confidence in detection of this point mutation (Fig. 2B).

### Gamma distribution model for state dwell time and optimization of assay conditions

The sensitivity and selectivity of this assay relies on the evaluation of the state dwell-time distribution of the SNV and WT molecules. The previously established Poisson model predicts that ~1 h of data acquisition is required to resolve this SNV if only the number of binding and dissociation events is considered. However, it is clear that single-nucleotide discrimination can be achieved within 10 min in this case (Fig. 2). We attempted to construct a more effective model to correlate binding kinetics to single-nucleotide selectivity. The distribution of state dwell time can be described by Gamma distribution^23^.


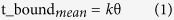



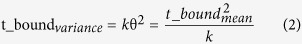


Where *k* is the shape parameter and θ is the rate parameter. In this case, *k* is the number of binding events during the acquisition time. *k* can be described by a Poisson distribution (see Supporting Information)^18^. For simplicity, we used the average of *k* for further calculation,


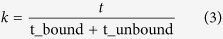


where *t* is the acquisition time. If τ_*bound*,*mean*_ for SNV and WT DNA molecules are separated by a given multiple of the standard deviation (*n*), *n* can be defined as discrimination capability factor.


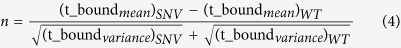


By substitution, the equation 4 can be rearranged to:


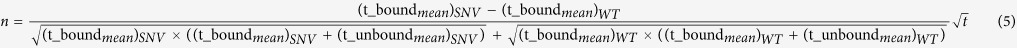


According to Equation 5, the discrimination capability factor is determined by the binding kinetics and data acquisition time. If SNV and WT are separated by a given multiple of the standard deviation (e.g. *n* = 3), then the calculated acquisition time is predicted to be ~16 min, given the assay conditions described in Fig. 1. First, acquisition time was optimized. As shown in Supplementary Fig. S2, 10 min of data acquisition is sufficient for SNV detection with high sensitivity and specificity, although n is slightly less stringent in this condition. Therefore, the acquisition time was fixed to 10 min.

**Figure 3:**
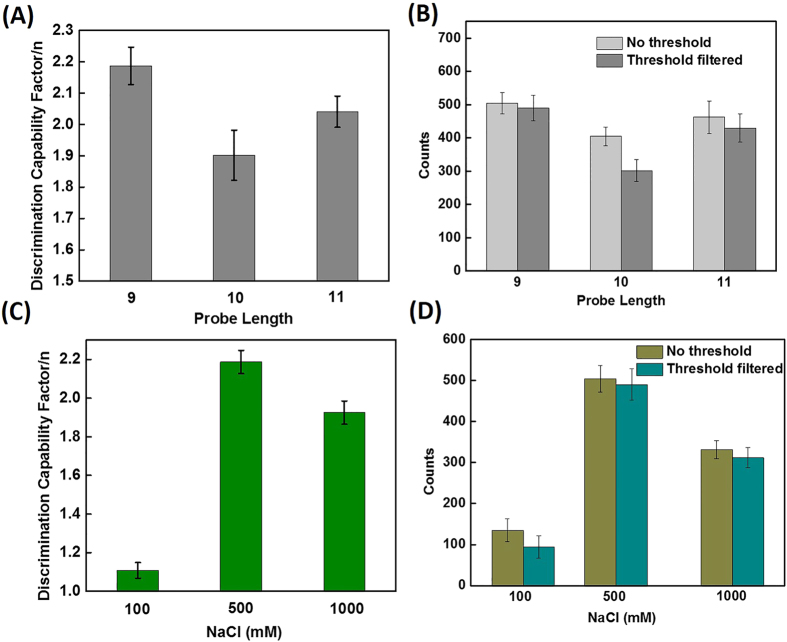
(**A**) Discrimination capability of probes with varying length. (**B**) Sensitivity comparison of probes with varying length. The thresholds used for 9-, 10-, and 11-nt probes were 6.8, 12.3, and 14.2 s, respectively. (**C**) Discrimination capability of 9-nt probe at different NaCl concentrations. (**D**) Sensitivity comparison of 9-nt probe with varying NaCl concentration. The thresholds used for 100, 500, and 1000 mM NaCl were 6.2, 6.8, and 11.2 s, respectively.

At a given temperature, DNA binding kinetics rely on the number of complementary bases and ionic strength^24^. Next, the probe length was optimized. We found that a 12-nt fluorescent probe is too long for transient binding, due to the high G-C content in the DNA sequence; the SNV and WT targets exhibited similar fluorescence trajectories (Supplementary Fig. S3). The 10- and 11-nt probes show similar fluorescent trajectories as the 9-nt probe (Supplementary Fig. S4). As shown in Fig. 3A, the discrimination capability factors (n) of the 10- and 11-nt probes are lower than the 9-nt probe, suggesting these probes have relatively lower single-nucleotide selectivity. As a consequence, they are less sensitive for SNV detection (Fig. 3B).

Because oligonucleotides are polyanionic, the addition of monovalent cations (e.g., Na^ + ^and K^ + ^) is known to enhance the binding strength between the two strands. We therefore investigated the effect of NaCl on the single-nucleotide discrimination capability by characterizing the binding kinetics as function of NaCl concentration. The dwell-time distributions of the 9-nt probe with 100 and 1000 mM NaCl are shown in Supplementary Fig. S5. The assay’s discrimination capability and sensitivity are lower at 100 and 1000 mM NaCl concentrations than at 500 mM (Fig. 3C and D). As expected, we found that the gamma distribution can be used to justify the optimal conditions that we determined empirically. In accordance with the above findings, we chose to use the 9-nt fluorescent probe with 500 mM NaCl for low-abundance mutation detection and to probe mutation in cancer cell lines.

The binding rate *k*_*on*_, the unbinding rate *k*_*off*_, and the dissociation constant *K*_*d*_ are shown in Supplementary Fig. S6. The *k*_*on*_ is derived from a regression line of pseudo-first order constant 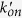 over the probe concentration (Supplementary Fig. S6A and D). *k*_*on*_ depends on temperature (*T*) and activation enthalpy (*H*_*a*_), as 
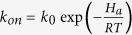
. As expected, *k*_*on*_ increases with increasing duplex length and NaCl concentration, because *H*_*a*_ decreases with increasing numbers of base pairs and NaCl concentration^24^. In fact, accurate calculation of *k*_*on*_ for short oligomers is relatively complicated. It has been reported that, for certain DNA special sequences, *k*_*on*_ slightly decreases with increasing duplex length^22^. *k*_*off*_ and *K*_*d*_ both show the reverse trend (Supplementary Fig. S6B,C,E, and F). Moreover, the *K*_*d*_ trends are consistent with the results predicted by NUPACK^25^ (Supplementary Fig. S6G and H). We also examined the correlation between DNA probes’ predicted melting temperature (T_m_) and binding kinetics, *k*_*on*_ (r = 0.931) and *k*_*off*_ (r = −0.934) (Supplementary Fig. S7), which was not clear from our previous work^18^. Clearly, duplexes with higher melting temperatures are more stable, leading to a fast binding rate and a slow unbinding rate. These observations can guide the design of transient binding probes.

### Detection of low-abundance point mutations using synthetic DNA

**Figure 4:**
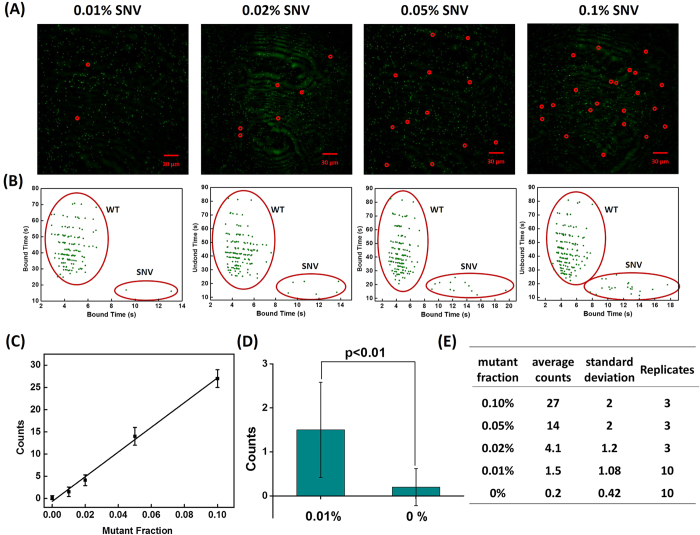
(**A**) Time-averaged fluorescence image consisting of fluorescent puncta formed by binding of probes to SNV and WT DNA molecules, as well as non-target background. Individual fluorescent puncta were analyzed for kinetics of probe binding to distinguish between signal from SNV and WT DNA molecules. The SNV molecules are highlighted by red circles. (**B**) SNV and WT target molecules exhibit distinct clusters classified by k-means clustering of bound time values. The SNV concentration was varied while the total target concentration remained fixed at 10 pM. The optimal assay conditions are as follows: probe concentration of 25 nM, NaCl concentration of 500 mM. (**C**) Linear relationship between mutant fraction and positive SNV counts. (**D**) Count numbers for a mutant: wild type frequency of 0.01% is significantly different from a 0% frequency (student *t*-test, *p* < 0.01). (**E**) The mean and standard deviation of SNV counts and number replicates for each mutant: WT frequency tested.

To test whether our method can detect SNVs at different variant allele frequencies (VAF), particularly at extremely low abundance, we first determined the detection limit of the KRAS c.34 A mutant. Our assay detected this mutation at concentrations as low as 1 fM, which is very sensitive for an amplification-free assay^26^,^27^ (Supplementary Fig. S8). Next, we demonstrated that the SNV can be clearly differentiated from the WT target at a mutant to WT frequency as low as 0.01%. By analyzing the fluorescence trace, we were able to identify individual SNV molecules in the time-averaged image (Fig. 4A). The dwell-time distribution reveals that SNV counts gradually increase with increasing abundance, even against a large background signal from WT DNA (Fig. 4B and C). The significant difference (p < 0.01) between 0.01 and 0% was noted (Fig. 4D). The average, standard deviation, and replicates of each mutant fraction are shown in Fig. 4E.

### Point-mutation detection in single-standed DNA reverse-transcribed from cellular mRNA in two cancer cell lines

To expand the generality of our method, we used our assay to investigate the point mutation BRAF V600E (c.1799 T > A), which is prevalent in many types of cancers^28^. Based on our observations from the probe design for KRAS c.34 G > A, we hypothesized that the hybridization kinetics and thermodynamics of the 9-nt probe in 500 mM NaCl can be used as reference for the design of other probes. We predicted the *K*_*d*_ of 9- and 10-nt BRAF c.1799 A probes under varying NaCl concentrations (100–1000 mM, with 100 mM increments) using NUPACK. As shown in Supplementary Fig. S9, the *K*_*d*_ of a 9-nt BRAF probe at 900 mM NaCl is most similar to the 9-nt KRAS probe at 500 mM NaCl. As expected, distinct WT and mutant fluorescence trajectories as well as WT and mutant clusters in the dwell-time map were found from the single-molecule assay (Supplementary Fig. S10). There were fewer BRAF wild type counts than KRAS wild type counts, which can be attributed to the larger free energy difference between the T-T mismatch versus the G-T mismatch in the WT-probe duplexes, relative to the T-A matched pair in the SNV-probe duplexes.

**Figure 5:**
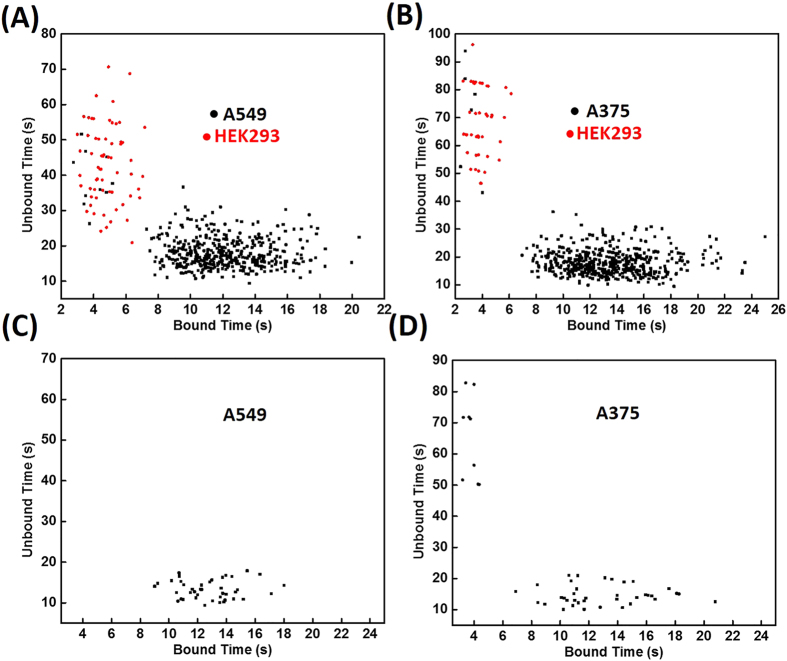
(**A,B**) Dwell-time distribution of single-molecule counting of KRAS c.34 G > A (**A**) and BRAF c.1799 T > A (**B**) mutation in different cell lines, using a probe that is fully complementary with SNVs. (**C,D**) Dwell-time distribution of single-molecule counting using a probe that is fully complementary with WT sequence. WT DNA sequences were found in the two cancer cell lines, A549 (**C**) and A375 (**D**). The concentrations of NaCl for KRAS and BRAF are 100 and 900 mM, respectively.

Next, we tested the feasibility of this method for point mutation detection in cellular mRNA from human cancer cell lines. Lung carcinoma cell line (A549) and melanoma cell line (A375) were chosen, because they have high incidence of KRAS and BRAF mutations, respectively. As a negative control, we also probed for KRAS and BRAF mutations in HEK-293 cells, which are predicted to only contain WT mRNAs. Total RNA was extracted from each cell line, and the corresponding cDNAs were then synthesized by reverse transcription. Standard PCR reactions were first carried out to quickly amplify the cDNA products. We generated single-stranded DNAs for single-molecule counting via asymmetric PCR using non-equivalent concentrations of primers. All of the PCR products were 80 nt in length. Gel analysis is shown in Supplementary Fig. S11. Both of the probes were 9 nt long, and the NaCl concentrations for KRAS and BRAF assay were 500 and 900 mM, respectively. The synthetic PCR amplicons were first tested in a single-molecule assay. As shown in Supplementary Fig. S12, the 80-nt single-stranded DNA exhibits a similar dwell-time distribution as the 39-nt model target, suggesting that the overall detection procedure is not affected by increasing DNA product length. Figure 5A and B summarizes the results of single-molecule counting assays for the targets from these cell lines. Mutant was found in the two cancer cell lines, while only the wild type gene was found in the normal (HEK 293) cell line. Interestingly, data points that represent the existence of mismatch between the probe and targets were also found in the two cancer cell lines. This can be attributed to the presence of some WT sequences or other types of mutations in the PCR products. We therefore synthesized fluorescent probes that are fully complementary with wild type targets. As shown in Fig. 5C and D, positive WT signal was found in the two cancer cell lines. The origin of these targets is not clear. First, genomic diversity within a single cell has been demonstrated by single-cell sequencing approaches^29^. Second, this result could also result from replication errors introduced by the Taq polymerase, which introduces one error per 3700 nucleotide incorporation events^30^,^31^. Third, RNA contamination or non-pure cell cultures can be a minor reason although we repeated the experiments carefully to reduce their probabilities. High-fidelity polymerases are better suited to post-amplification analysis. To differentiate between these hypotheses, more studies, such as single-cell sequencing and amplification with high-fidelity polymerases, are needed.

## Discussion

The single-molecule approach presented here addresses some of the current limitations in the field of point-mutation detection. The single-nucleotide selectivity of hybridization-based probes was greatly improved by utilizing the transient binding of short probes and by determining the binding kinetics. The major advantage of our single-molecule platform is its unique ability to resolve kinetic rates of binding by individual units in a digital manner. This allows us to directly identify SNVs at single-molecule level and detect SNVs at a relative abundance as low as 0.01%. This sensitivity is better than some approaches that use fluorescence probes^20^ and electrochemical sensors^32^. Our method’s ability to discriminate single nucleotides is comparable with existing droplet digital PCR approaches, which have an SNV abundance detection limit ranging from 0.001% to 0.25%^3^,^33^ with a detection limit of 0.1% for high-confidence detection^34^. Moreover, our method has significant advantages over conventional next-generation sequencing (NGS) for the detection of known point mutations in assay time and detection limit. Conventional NGS can only detect mutant DNA at 1–2% mutant to WT frequency, and is also affected by the sequencing depth^35^. A newly developed NGS approach permits detecting point mutations at low abundance with high confidence benefiting from the “duplex sequencing” strategy^36^. The “duplex sequencing” used in this approach greatly reduces errors by independently tagging and sequencing each of the two strands of a DNA duplex allowing an extremely low error rate which overcomes the limitation of high error rate caused by polymerase in conventional NGS.

Although previously developed technology can detect single nucleotide changes^18^, the approach we describe here provides superior quantification of point mutations. We propose a new model, which is more suitable for the optimization of assay conditions, and also demonstrate the ability to detect rare mutant alleles, as well as the feasibility to detect mutant mRNA from cancer cell lines. The gamma distribution model of state dwell time holds great potential for single-molecule studies with various types of reversible binding.

This method still has limitations. For example, the requirement for single-stranded nucleic acid prohibits the direct testing of genomic DNA. This method’s dependence on conventional PCR can lead to detection bias, which is a potential problem for multiplexing. In our analysis of cancer cell lines, we found data points representing probe-target mismatches, which are probably caused by polymerase errors during amplification. This could be improved by adapting the amplification strategy used for duplex sequencing in the advanced NGS approach mentioned above^36^. Looking ahead, the transient binding tag can become a promising signal reporter in single-molecule analysis for heralding broad applications.

## Methods

### Materials and Chemicals

All of the oligonucleotides used in this work were synthesized and purified by HPLC from Sangon Co. (Shanghai, China) and their sequences are listed in Table S1. (3-Aminopropyl) triethoxysilane (APTES), 3,4-dihydroxybenzoate (PCA), protocatechuate dioxygenase (PCD) and Trolox were from Sigma-Aldrich (St. Louis, MO). mPEG-succinimidyl valerate (mPEG-SVA, MW, 5000), and biotin‐PEG‐ succinimidyl valerate (biotin-PEG-SVA, MW, 5000) were purchased from SeeBio Co. (Shanghai, China). All chemicals were used as received without additional purification. A PicoPure^®^ RNA Isolation Kit was obtained from Thermo Fisher (Fremont, CA). The reverse transcription system (A3500) was from Promega (Fitchburg, WI). Taq DNA polymerase was purchased from NEB (Beverly, MA). HEK-293, A549, and A375 cell lines were obtained from ATCC (Manassas, VA). DNase/RNase-free deionized water from Tiangen Biotech Co. (Beijing, China) was used in all experiments.

### Total internal reflection fluorescence microscope setup

A quartz slide or coverslip was coated with a 10:1 mixture of mPEG and biotin-PEG prior to construction of the sample cell as previously described^37^. Sample cells were constructed by cutting a piece of a yellow pipet tip (1 cm in length, Axygen) and fixing it to a coverslip with epoxy adhesive. Prepared slides were stored in the dark for up to 1 week. Single-molecule counting experiments were performed using an Olympus IX-83 objective-type TIRF microscope equipped with a 60 × oil-immersion objective (APON 60XOTIRFM, 1.49NA), Cell^TIRF and z-drift control modules, as well as an EMCCD camera (IXon 897, Andor, EM gain 1000), were used for all measurements.

### Synthetic oligonucleotides solution

All solutions were prepared in 1.7-mL microcentrifuge tubes, and target oligonucleotides were diluted in the presence of 0.03 mg/mL polyT as a carrier in order to protect the targets from absorbing on the tube. For low-abundance mutation detection assays, SNV and WT DNA were mixed in varying ratios, while keeping the total target concentration fixed at 10 pM.

### Single-molecule counting of synthetic oligonucleotides

The slide surface was briefly incubated with 100 μL TE buffer (10 mM Tris-HCl, 1 mM EDTA, pH 8.0) followed by 20 μL 1 mg/mL streptavidin for 10 min. Then, excess streptavidin was flushed out using TE buffer. Next, 20 nM of the biotinylated DNA capture probe (in 1 × PBS buffer) was added for 10 min, and the excess flushed out by rinsing with 1 × PBS three times. Target oligonucleotide solution was introduced into the sample cell and incubated for 30 min. This long incubation time for the objective-type TIRF microscopy measurements was necessary, because there is a time delay as analytes are transported to the imaging surface. After flushing out the target solution with 1 × PBS, the following solutions were added to the sample cell: (1) an imaging buffer containing 10 mM phosphate buffer (no NaCl), (2) an oxygen-scavenging system consisting of 2.5 mM PCA, 25 nM PCD, and 1 mM Trolox^38^, (3) 25 nM of the fluorescently labeled probe, and (4) NaCl at varying concentrations. The transient binding of probes to target molecules was monitored under illumination by 532 nm laser light. Image acquisition was performed at a rate of 2 Hz using the EMCCD camera. Note that for single molecule assays, the room temperature was maintained at 25 °C.

### Analysis of single-molecule fluorescence data

Fluorescence time trajectory was extracted from acquired movies by custom MATLAB code. The hidden Markov model (HMM) is a stochastic model that maps measured values to unobserved (or hidden) states. The trajectories were fitted with HMM using QuB software to identify the number of transitions and dwell times of the bound (τ_bound_) and unbound (τ_unbound_) states for each candidate molecule. Based on control measurements in the absence of target, a universal threshold of transitions (12 times) and minimal τ_bound_ (3 s) and τ_unbound_ (3 s) were used to remove the background molecules from the candidates.

### PAGE gel analysis of PCR products

PCR products and synthetic oligos (final concentration 200 nM) were separated on a native 20% (w/v) polyacrylamide gel. The gel was stained with SYBR gold (Life Technologies) and imaged on a gel transilluminator (ThermoFisher Scientific).

### Cellular RNA extraction, reverse transcription, and asymmetric PCR

Cellular total RNA extraction was performed by using a PicoPure^®^ RNA Isolation Kit, which yields high recovery of total cellular RNA. Cells were incubated with extraction buffer at 42 °C for 30 min, followed by centrifugation at 800 g for 2 min. Then the cell extract and ethanol were loaded into the pre-conditioned purification column. Next, the column was centrifuged for 2 min at 100 g immediately followed by a centrifugation at 16,000 g for 30 seconds to remove the flowthrough. 100 μL Wash Buffer 1 was added into the purification column, followed by a centrifugation for one minute at 8,000 g. 1 U of DNase was added and incubated at 37 °C for 20 min to degrade DNA. Then the column was rinsed with two volumes of Wash Buffer 2. cDNAs were then prepared by reverse transcription using a commercially available reverse transcription kit (Promega, CAS: A3500). The reverse transcription system consisted of 4 μL MgCl_2_, 2 μL 10 × reverse buffer, 2 μL dNTP, 0.5 μL RNase inhibitor, 15 unit AMV enzyme, 1 μL Oligo (dT)15, and 1 μL isolated total RNA. The program was set as the following: 42 °C for 15 min, 95 °C for 5 min, 4 °C for 5 min. Next, the cDNAs were amplified by standard PCR followed by asymmetric PCR. The reagents used for standard PCR are shown in Table S2. The amplification program (94 °C for 30 s, 56 °C for 30 s, 72 °C for 20 s; 30 cycles) was performed on a Rotor-Gene Q5 plex HRM Instrument. The standard PCR product was diluted to 2 ng/μL for asymmetric PCR, where the forward primer and reverse primer concentrations were adjusted to 2 μM and 0.2 μM respectively (all other reagents were the same as described for standard PCR). The same thermocycler program was used for asymmetric PCR as for standard PCR. The final PCR product was diluted to 200 fM in 1 × PBS for single-molecule assays.

## Additional Information

**How to cite this article:** Su, X. *et al*. Single-Molecule Counting of Point Mutations by Transient DNA Binding. *Sci. Rep.* **7**, 43824; doi: 10.1038/srep43824 (2017).

**Publisher's note:** Springer Nature remains neutral with regard to jurisdictional claims in published maps and institutional affiliations.

## Supplementary Material

Supporting Information
