# Supplementary material for: Single-Molecule Counting of Point Mutations by Transient DNA Binding
Source: Sci Rep. 2017 Mar 6;7:43824. doi: 10.1038/srep43824 (PMC5338343; doi:10.1038/srep43824)
Supplement: Supporting Information [file srep43824-s1.pdf]

Supplementary Information

Single Molecule Counting of Point Mutations by Transient DNA Binding

Xin Su[1], Lidan Li[1], Shanshan Wang[2], Dandan Hao[1], Lei Wang[1], and Changyuan Yu[1,*]

[1]College of Life Science and Technology, Beijing University of Chemical Technology, Beijing, China, 100029.

[2]Institute of Quality Standard and Testing Technology for Agro-Products, Chinese Academy of Agricultural Sciences, Beijing 100081, China.

*corresponding. yucy@mail.buct.edu.cn

Table S1 Synthetic oligonucleotide used in this work

Name	Sequance (5'-3')
Strands for single molecule assays	
KRAS c.34G	GACTGAGTATAAACTTGTGGTAGTTGGAGCTGGTGGCGT
KRAS c.34A	GACTGAGTATAAACTTGTGGTAGTTGGAGCTAGTGGCGT
Capture probe for KRAS	AACTACCACAAGTTTATACTCAGTC-Biotin
12-nt Fluorescent probe for KRAS	Cy3-GCCACTAGCTCC
11-nt Fluorescent probe for KRAS	Cy3-GCCACTAGCTC
10-nt Fluorescent probe for KRAS	Cy3-CCACTAGCTC
9-nt Fluorescent probe for KRAS	Cy3-CCACTAGCT
BRAF c.1799T	TAAAAATAGGTGATTTTGGTCTAGCTACAGTGAAATC
BRAF c.1799A	TAAAAATAGGTGATTTTGGTCTAGCTACAGAGAAATC
Capture probe for BRAF	GCTAGACCAAAATCACCTATTTTTA-Biotin
9-nt Fluorescent probe for BRAF	Cy3-TTTCTCTGT
9-nt Fluorescent probe for KRAS wild type	Cy3-CCACCAGCT
9-nt Fluorescent probe for BRAF wild type	Cy3-TTTCACTGT
Strands for PCR	
KRAS forward primer	TTATAAGGCCTGCTGAAAAT
KRAS reverse primer	TCCGTTCTCAGGAACTGCTA
BRAF forward primer	TCTTCATGAAGACCTCACAG
BRAF reverse primer	TGCTACCTACCCTAGGGTCG

Bases highlighted in blue are the mutation allele studied.

Table S2 Normal PCR reaction protocol

Component	Amount
10× Thermopol Buffer	5 μL
$MgCl_2$, 25 mM	1 μL
dNTP Mixture, 10 mM	1 μL
Forward Primer, 10 μM	1 μL
Reverse Primer, 10 μM	1 μL
Taq Polymerase	1.25 units
First strand cDNA reaction	10 μL
ddH_2O	Fill to 50 μL

Shape parameter description in Gamma distribution.

k is the shape parameter in the Gamma distribution and is defined as the expected number of transitions between the bound and unbound state per unit time. We have

$$k = \frac{2k'_{on}k_{off}}{k'_{on}+k_{off}} \quad (1)$$

k'_{on} is the pseudo-first order rate of binding and k_{off} is rate of unbinding. In our previous work, k can be modeled by Poisson distribution.

Supporting Figures

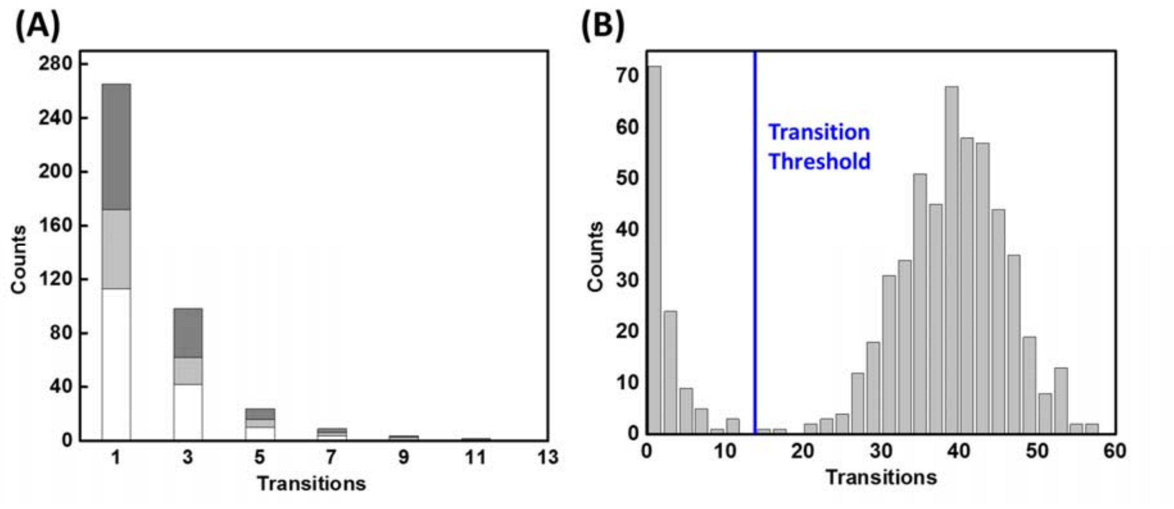

Fig. S1 A) Transition histogram in absence of target DNA sequence for triplicate measurements. Fluorescence state change was as transitions. B) Transition histograms in presence of 200 fM ssDNA target. According to the transition distribution of the background (in the absence of targets), the transition threshold was set as 12. Additionally, to rule out the fluorescence bursts, the minimal t_bound and t_unbound were set as 3 s. This is the universal threshold for all assays to remove non-nucleic acid background.

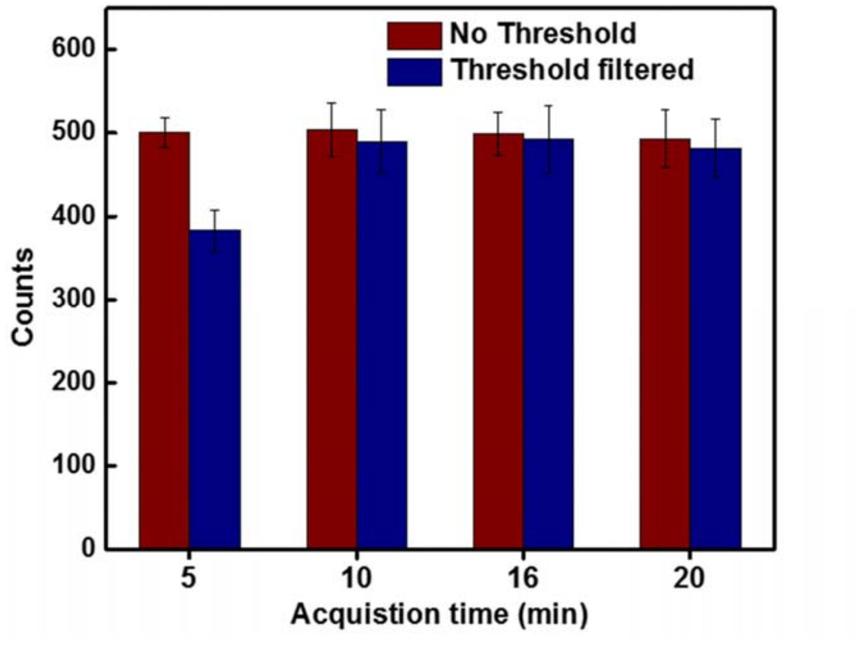

Fig. S2 Sensitivity comparison of 9-nt probe with different acquisition times.

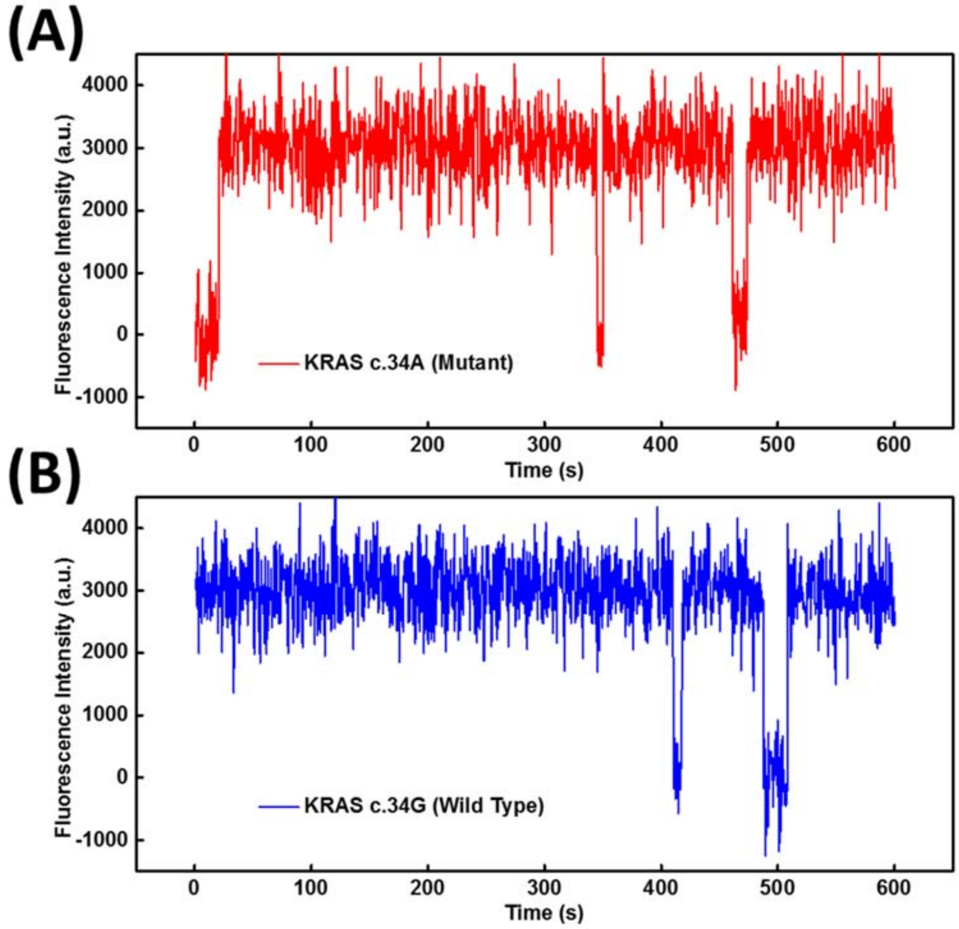

Fig. S3 Fluorescence time trajectory of (A) KRAS mutant, (B) KRAS wild type with 12 nt fluorescent probe at 500 mM NaCl under 10 min acquisition time where the probe and target concentrations were 25 nM and 200 fM, respectively. Due to the high G-C content in this sequence, the binding is close to stable hybridization. The wild type and mutant can't be discriminated.

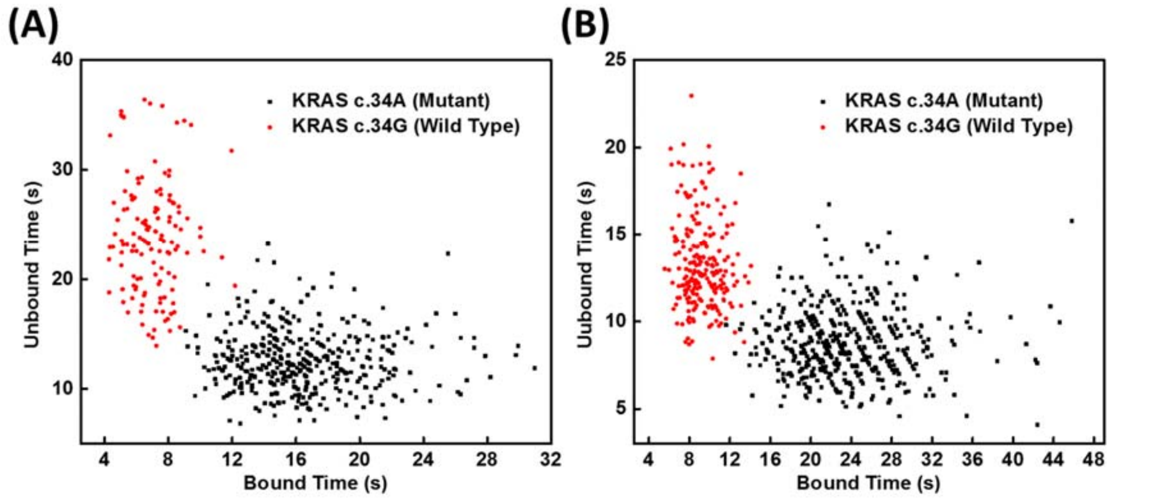

Fig. S4 State dwell time distribution of KRAS wild type and mutation by using 10 nt (A) and 11 nt (B) probe. The NaCl concentration is 500 mM.

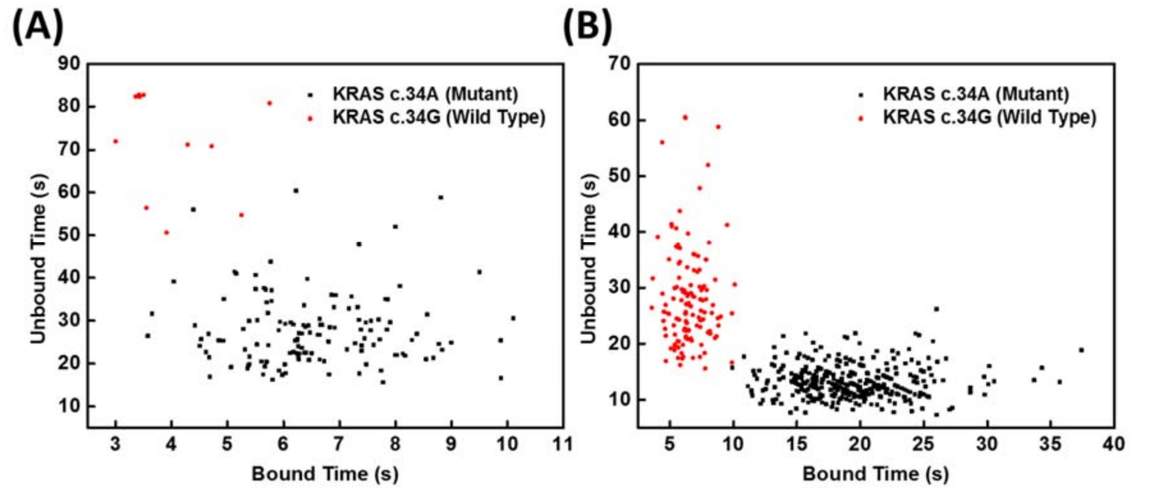

Fig. S5 State dwell time distribution of KRAS wild type and mutation by using 9 nt probe with 100 mM (A) and 1000 mM NaCl (B).

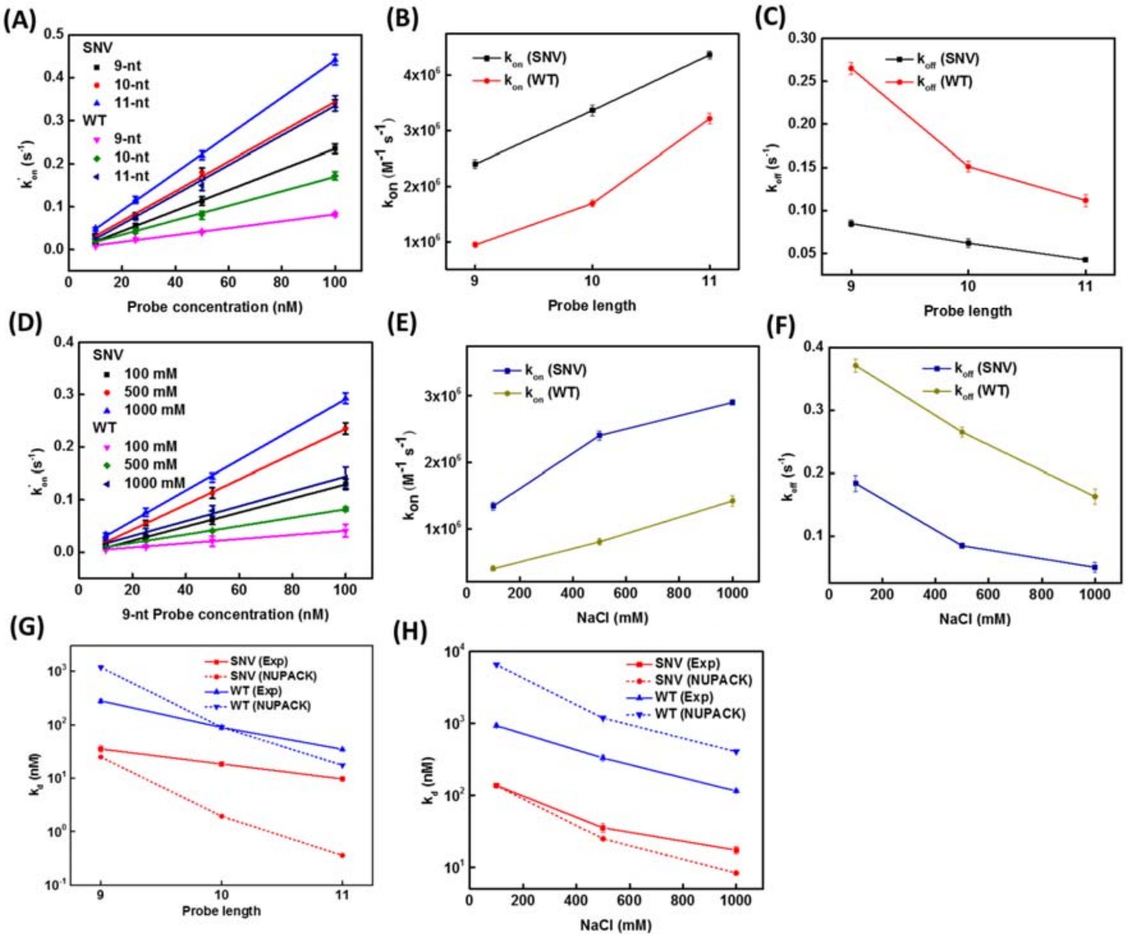

Fig. S6 Transient binding kinetics and thermodynamics. (A and D) Stern-Volmer plots of pseudo-first order constant k'_{on} for different assay conditions, illustrating the expected linear dependence of k_{on} with probe concentration. The slopes represent binding rate k_{on}. (B and C) Binding rate k_{on} and unbinding rate k_{off} as a function of probe length. (E and F) Binding rate k_{on} and unbinding rate k_{off} of 9-nt probe as a function of NaCl concentration. (G) Predicted and experimental dissociation constant K_d as a function of probe length. (H) Predicted and experimental dissociation constant K_d of 9-nt probe as a function of NaCl concentration.

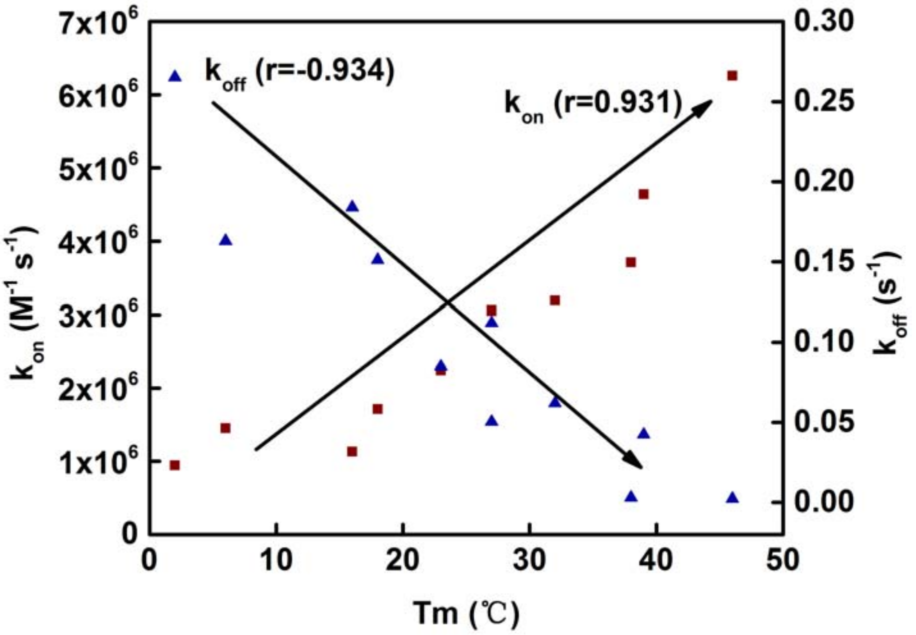

Fig. S7 The correlation between hybridization kinetics and predicted T_m (NUPACK).

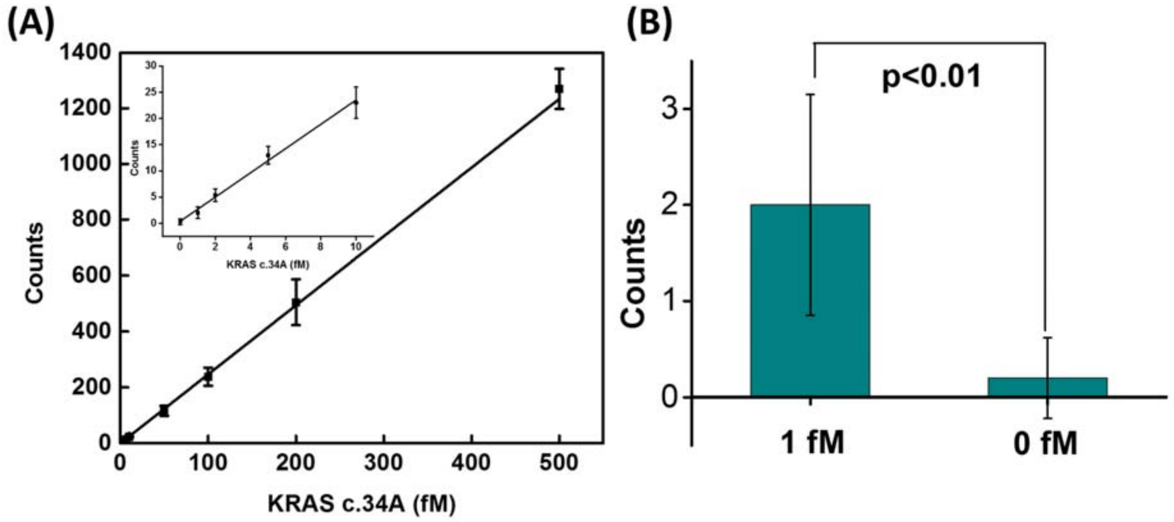

Fig. S8 (A) Standard curves from single molecule assays of target KRAS c.34A. Linear fits were constrained to a y-intercept of 0, yielding R^2 =0.996. Note that the count number was obtained using "stringent threshold". Inset: Standard curves of the concentrations below 50 fM. The LOD is defined as 3 × standard deviation of the background. (B) Significant (P < 0.01) differences between 1 fM and 0 fM is noted.

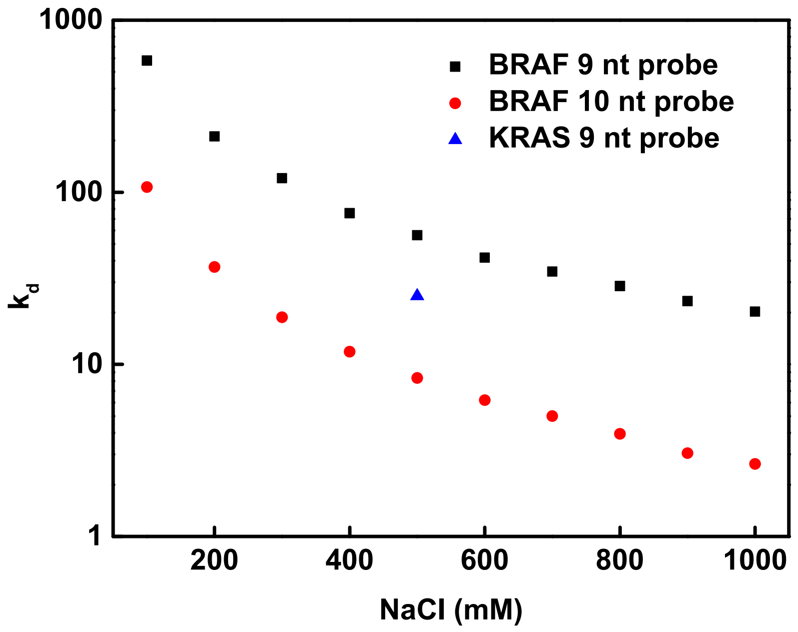

Fig. S9 Dynamic equilibrium prediction of BRAF c.1799A mutation with 9 or 10-nt probes which were completely complementary with BRAF c.1799A based on NUPACK.

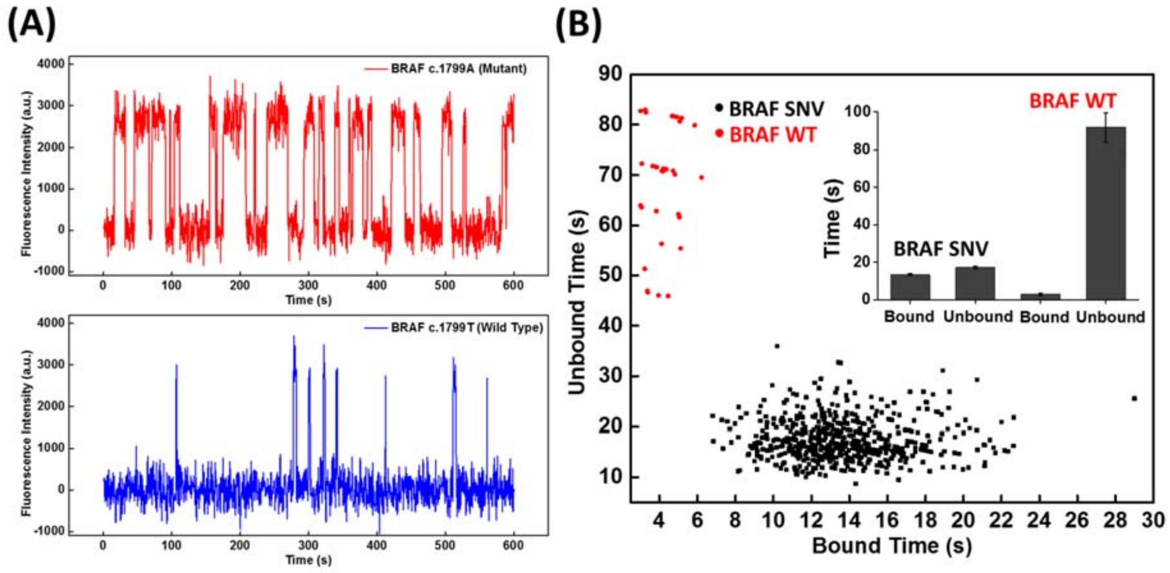

Fig. S10 A) Fluorescence time trajectory of BRAF SNV and wild type with 9 nt fluorescent probe where the probe and target concentrations were 25 nM and 200 fM, respectively. NaCl was 900 mM. B) State dwell time map of BRAF SNV and WT. Inset: the average bound and unbound time of SNV and WT.

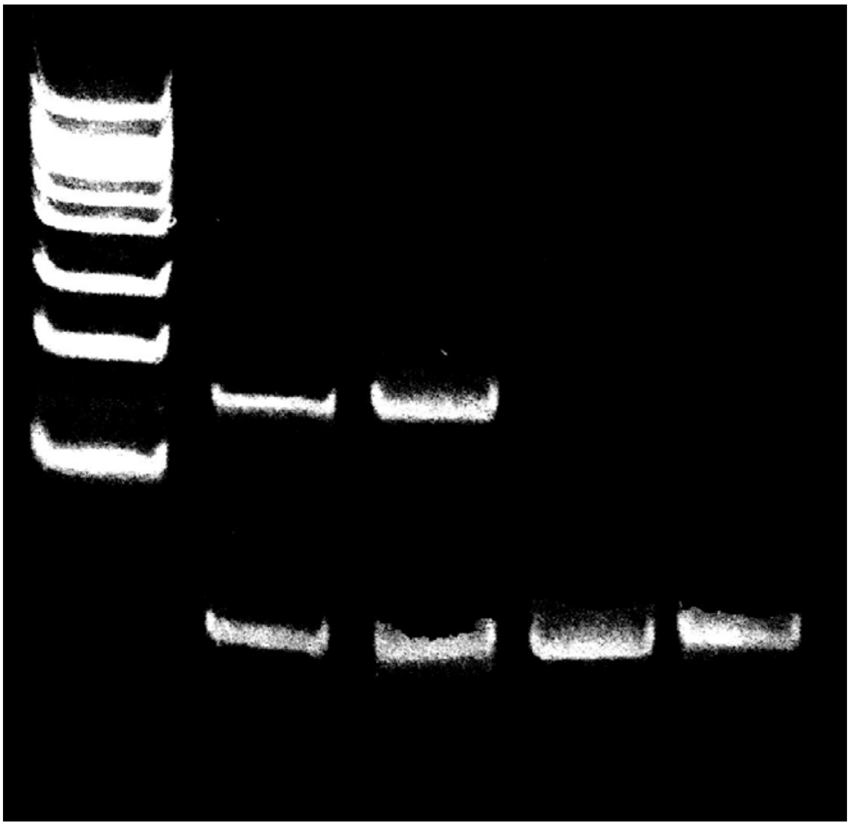

Fig. S11 PAGE gel analysis of the PCR products. Lane1: 50 bp DNA marker. Lane2: Asymmetric PCR products of KRAS gene from A549 cell line. Lane3: Asymmetric PCR products of BRAF gene from A375 cell line. Line 4: Synthetic ssDNA of KRAS amplicon. Line 5: Synthetic ssDNA of BRAF amplicon.

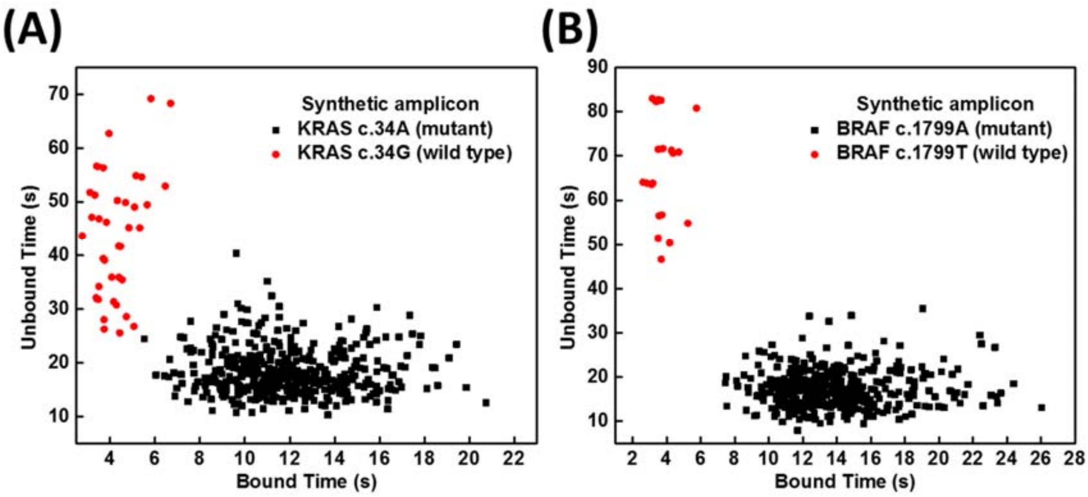

Fig. S12 Single molecule assay for synthetic PCR amplicons (80-nt), KRAS (A) and BRAF (B). The assay conditions are the same with the 39-nt model strands.
